# Obituary

**Published:** 1917-02

**Authors:** 


### OBITUARY

HENRY W. HARVEY, D.D.S. Dr. Harvey, one of the best known practitioners of Battle Creek, died in a sanitarium at Waukesha, Wis. Dr. Harvey had been in precarious health for several years, and made a strong fight to regain his health. He graduated from the College of Dental Surgery of the University of Michigan in 1899. He served as clinical demonstrator one year and then located in Battle Creek, where he built a good practice, and made a fine place for himself as a highly respected citizen. He was a strictly honorable and patriotic citizen, and was loyal to every good cause in his city and the state. His popularity caused him to over work, and his none too rugged constitution failed him three or four years ago, and he never recovered sufficient health to prevent the final breakdown. He will be remembered by a host of friends, both in and out of the profession. He was in his forty-fifth year when he died.

